# Impact of platelet-rich fibrin on mandibular third molar surgery recovery: a systematic review and meta-analysis

**DOI:** 10.1186/s12903-019-0824-3

**Published:** 2019-07-25

**Authors:** Xu Xiang, Ping Shi, Ping Zhang, Jun Shen, Jian Kang

**Affiliations:** 1 Department of oral and maxillofacial surgery, Tianjin Stomatological Hospital, Tianjin, 300041 China; 2 Department of periodontics, Tianjin Stomatological Hospital, Tianjin, 300041 China

**Keywords:** Platelet-rich fibrin, Mandibular third molars, Systematic review, Meta-analysis

## Abstract

**Background:**

The present study investigated and evaluated the efficacy and safety of platelet-rich fibrin (PRF) in patients during bilateral mandibular third molars extraction by systematic review and meta-analysis.

**Methods:**

The PubMed, Embase, and Cochrane library databases were retrieved, and the effect of PRF on the healing process of the alveolar socket after surgical extraction of the mandibular third molars was evaluated by meta-analysis. The postoperative pain, swelling, trismus, osteoblastic activity, and soft tissue healing were assessed, and the incidence of alveolar osteitis, weighted mean difference (WMD)/standard mean difference (SMD), the risk ratio (RR), and the 95% confidence interval (CI) were calculated.

**Results:**

The current results showed that the local application of PRF during lower third molar extraction prevented postoperative complications. Subsequently, the pain (SMD = − 0.53, 95% CI: − 1.02–-0.05, *P*_heterogeneity_ = 0.001, *I*^*2*^ = 75.7%) and swelling (WMD = − 0.55, 95% CI: − 1.08–-0.01, *P*_heterogeneity_ = 0.573, *I*^*2*^ = 0) were relieved and the incidence of alveolar osteitis was reduced (RR = 0.35, 95% CI: 0.16–0.75, *P*_heterogeneity_ = 0.597, *I*^*2*^ = 0%). However, no significant difference was observed in trismus, osteoblastic activity, and soft tissue healing between the PRF and non-PRF groups.

**Conclusion:**

The current study confirms that PRF only reduces some of the postoperative complications but does not prevent all the postoperative complications. PRF significantly relieved the pain and swelling and reduced the incidence of alveolar osteitis after the extraction of an impacted lower third molar.

**Electronic supplementary material:**

The online version of this article (10.1186/s12903-019-0824-3) contains supplementary material, which is available to authorized users.

## Background

In oral surgery, the operation of the impacted third molar is one of the most common surgical procedures performed by oral and maxillofacial surgeons [1]. After the impacted third molars are removed in the early postoperative stage, patients usually present complications such as pain, swelling, and trismus [2, 3]. These inflammatory complications are crucial for patients and surgeons in order to develop the customized strategy for reducing the risk of complications and improving postoperative healing [4]. Several attempts using platelet-rich plasma administration, preoperative and postoperative antibiotics, cryotherapy, wound draining, the use of different kinds of flaps, and osteotomy using high- or low-speed rotary instruments, postoperative ice packs, analgesics, corticosteroids, and laser have been made to reduce the postoperative outcome of the removal of the third molar post-surgery [5–9].

Platelet-rich fibrin (PRF) is a novel strategy for concentrating the platelets (the preparation process without thrombin), which can be used for the enhancement after tooth extraction and residual cyst bone formation and promotion of the wound epithelialization [10–14]. The PRF originates from the slow, gradual polymerization occurring during centrifugation [15]. This is the second generation of immune platelet concentrate, collected as single fiber membrane protein components of the blood sample. These components are utilized for healing and immune regulation, especially, fibrin matrix in which, growth factors (vascular endothelial growth factor (VEGF), transforming growth factor (TGF)-A1, platelet-derived growth factor (PDGF)-AA, and insulin-like growth factor 1, leukocytic cells, and their cytokines such as, interleukin (IL)-4, IL-6, IL-1A, and tumor necrosis factor (TNF)) are enmeshed [10–14].

PRF is widely used for mandibular third molar surgery; however, its effect on potential post-surgical complications is unclear. The efficiency of local application of PRF to control the postoperative complications after the extraction of an impacted lower third molar has been investigated by several meta-analyses. Two previous meta-analyses conducted by Al-Hamed et al. [16] and Canellas et al. [17] had limitations since only two randomized controlled trials (RCTs) were included in the quantitative synthesis that compared the relevant interventions. Recently, He et al. [18] conducted a systematic review and meta-analysis to evaluate the efficacy of PRF on a mandibular third molar. These meta-analyses were followed by several RCTs on the same topic; however, the findings were controversial and no updated meta-analysis is yet available. Herein, we identified the eligible studies [19–21] and performed a detailed analysis at different time points. The present systematic review and meta-analysis investigated and assessed whether PRF was effective and safe for patients during the extraction of bilateral mandibular third molars.

## Methods

This study was designed in compliance with the guidelines of the 2009 Preferred Reporting Items for Systematic Reviews and Meta-Analysis (PRISMA) statement [22].

### Search strategy

The potentially relevant studies were identified by searching Pubmed, Embase, and the Cochrane library. A systematic and comprehensive search was performed on the three databases using a combination of keywords and medical subheadings: “platelet-rich fibrin” or “PRF”, “oral surgery”, and “third molar”(Additional file 1: Table S1). Alternative spellings and abbreviations were also considered. To identify additional studies, the reference lists of the included studies and relevant reviews were also searched manually. The literature search was limited to the English language, and the last search was performed on September 3, 2017 by two authors, independently, using a standardized approach. Any inconsistencies between the two authors were settled by group discussion to achieve a consensus.

### Selection criteria

The inclusion criteria included: 1) patients with bilateral mandibular third molars required surgical extraction; 2) at least two comparison groups: one group received PRF at the mandibular third molar and the other group received control treatment without PRF; 3) published in the English literature; 4) outcomes: alveolar osteitis, osteoblastic activity, pain, swelling, trismus, and soft tissue healing. The exclusion criteria were as follows: 1) the inclusion criteria were not fulfilled; 2) studies on the same population or overlapping database.

### Data extraction and quality assessment

The available data were extracted from each study by two investigators, independently, according to the inclusion criteria listed above; any disagreement was subsequently resolved by discussion with a third author. The following data were collected from each study: first author’s name, publication year, a country where the research was performed, number of patients, the gender of patients, mean age of the patients, time of follow-up, study design, and the outcomes. The quality of the RCTs was evaluated using the Cochrane Collaboration’s tool for assessing the risk of bias [23]. The assessment included the following components: random sequence generation, allocation concealment, blinding of patients, study personnel, blinding of outcome assessment, completeness of the outcome data, selective reporting of outcomes, and the other threats to validity (i.e. intention-to-treat analysis and completeness of follow-up). All these domains can be rated as either high, low, or unclear. Quality of evidence was assessed across important outcomes using GRADE approach to support management recommendations by the GRADEpro software (version 3.6). The criteria were based on study design, limitations, inconsistency, indirectness, imprecision, and other considerations. The quality of evidence was rated as high, moderate, low, or very low.

### Statistical analysis

We calculated the weighted mean difference (WMD) (continuous variables with same unit)/standard mean difference (SMD) (continuous variables with different unit) and 95% confidence intervals (CIs) for the continuous data, and the risk ratio (RR) and 95% CIs were calculated for dichotomous data. The heterogeneity of the studies was assessed using the Cochran’s Q test [24] that was quantified by the *I*^*2*^ statistic (considered as high heterogeneity for *I*^*2*^ > 50%). Preliminary analysis was conducted using a fixed-effects model (Mantel–Haenszel method) [25]; in the case of high heterogeneity, a random effects model was employed (Der Simonian and Laird) [26]. The relative influence of each study on the pooled estimate was assessed by excluding each study sequentially for sensitivity analysis. The publication bias was evaluated by visual inspection of the symmetry of the funnel plot and assessment of Begg’s and Egger’s test (*P* < 0.05 is representative of statistical significance) [27]. Statistical analyses were conducted using STATA software, version 12.0 (STATA Co., College Station, TX, USA), and all tests were two-sided.

## Results

### Characteristics of the studies

**Fig. 1:**
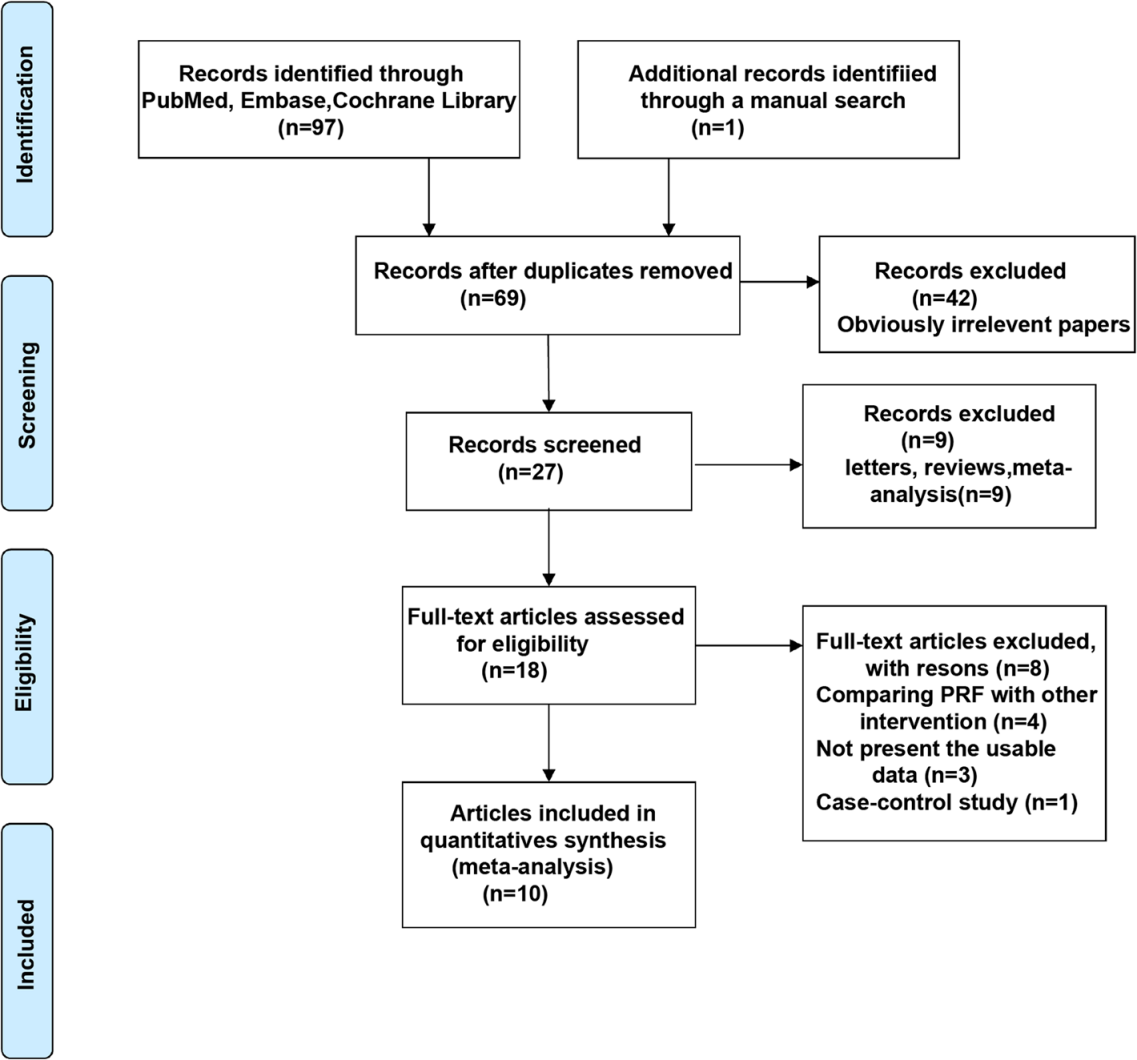
Schematic representation of the identification of the studies

**Fig. 2:**
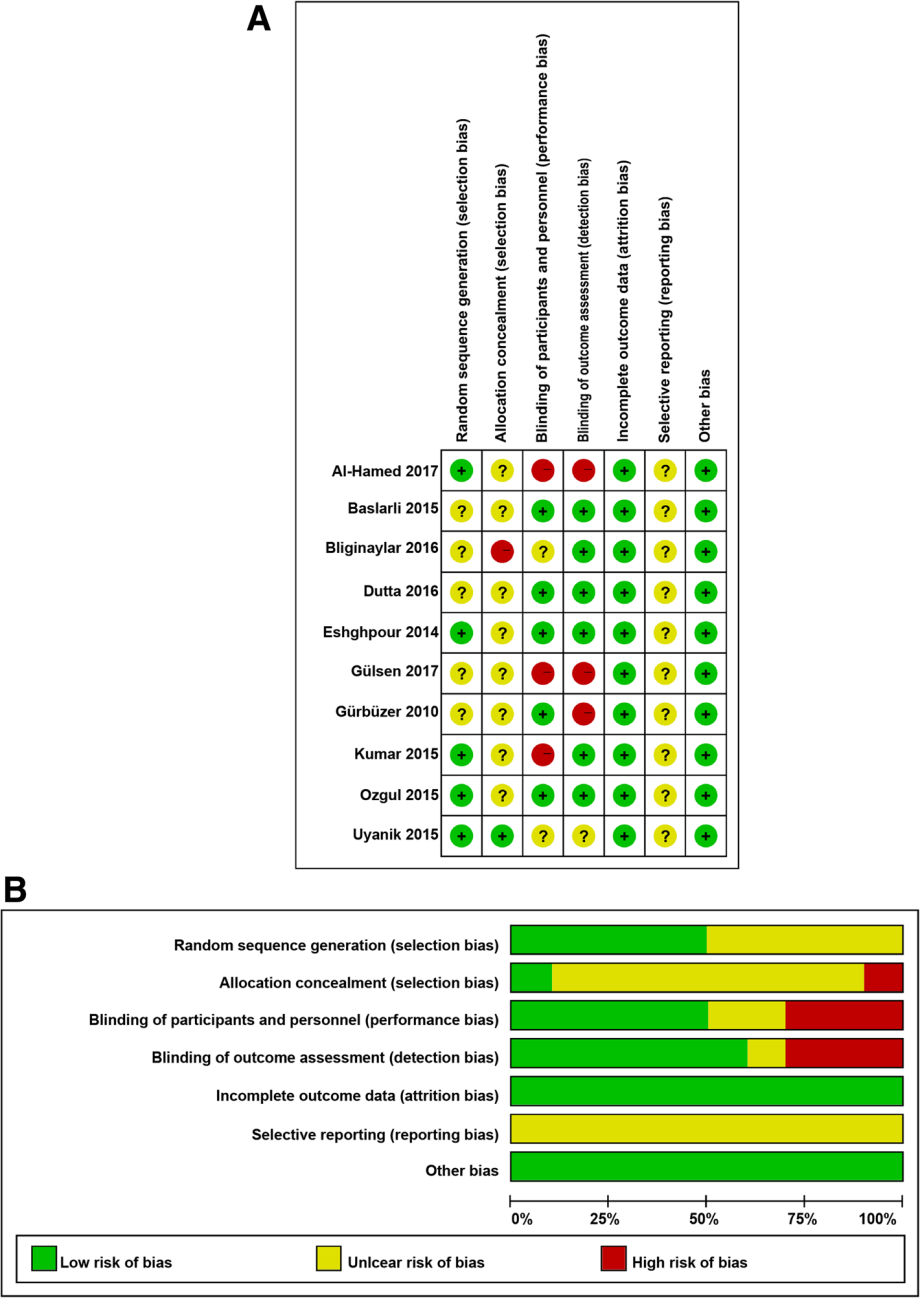
Risk of bias assessment for the randomized trials included in the meta-analysis. **a** Risk of bias summary; **b** Risk of bias graph. *Symbols*. (+): low risk of bias; (?): unclear risk of bias; (−): high risk of bias

A total of 98 articles were identified from the databases and manual search as described above. After excluding the duplicates, 69 articles were remaining. Subsequently, we evaluated the remaining articles and 42 were discarded because of irrelevance. Of the remaining 27 articles, 9 were excluded as they were letters, reviews, and meta-analysis. The remaining 18 full-text articles were assessed for potential eligibility, of which, 4 were excluded for comparing the PRF with other interventions, 3 were without usable data, and 1 was a case-control study. Finally, a total of 10 studies [19–21, 28–34] fulfilled the inclusion and exclusion criteria in this systematic review and meta-analysis (Fig. 1). The main characteristics of the eligible studies are summarized in Table 1. These 10 studies were also assessed qualitatively using the tools recommended by the Cochrane Collaboration for the risk of bias. A graph and summary of selection bias, performance bias, detection bias, attrition bias, reporting bias, and other biases identified in each study are shown in Fig. 2a and b. A previous study [34] had a high risk of bias in allocation concealment, 3 studies [20, 21, 31] had a high risk of bias in blinding of participants and personnel, and 3 studies [20, 21, 28] had a high risk of bias in blinding of outcome assessment. The quality of the evidence of each result was shown in Table 2. The evidence was graded as ‘moderate quality’ for swelling, ‘low quality’ for pain, alveolar osteitis, and soft tissue healing, and ‘very low quality’ for trismus and osteoblastic activity. The quality of evidence was downgraded to ‘moderate’, ‘low’ or ‘very low’ mainly due to high risk of performance bias (randomization and blinding), inconsistency (significant heterogeneity) and imprecision (pooled results included no effects).

**Table 1:** Characteristics of the studies included in this meta-analysis

Author/year of publication	Country	Gender	Mean age (Y)	Intervention		Follow-up (d)	Study design	Outcomes assessed
				PRF	Control			
Gürbüzer/2010 [28]	Turkey	7 males and 7 females	24.92 ± 4.69Y	14	14	28d	RCT, split-mouth	Osteoblastic activity
Eshghpour/2014 [29]	Iran	33 males and 45 females	25.09 ± 4.25Y	78	78	2 and 7d	RCT, split-mouth	Alveolar osteitis
Baslarli/2015 [30]	Turkey	7 males and 13 females	23.9Y	20	20	30 and 90d	RCT, split-mouth	Alveolar osteitis, osteoblastic activity
Kumar/2015 [31]	India	NA	PRF:25.25 ± 4.2Y Control:27 ± 5.27Y	16	15	90d	RCT	Trismus
Ozgul/2015[32]	Turkey	23 males and 33 females	NA	56	56	1,3, and 7d	RCT, split-mouth	Pain, swelling
Uyanık/2015 [33]	Cyprus	4 males, 6 females	22.65Y	10	10	1,2,3, and 7d	RCT, split-mouth	Pain, swelling, trismus
Bilginaylar/2016 [34]	Cyprus	22 males and 37 females	PRF:21.75Y Control:22.5Y	40	40	1,2,3, and 7d	RCT	Pain, swelling, trismus
Dutta/2016 [19]	India	27 males and 13 females	27 ± 5Y	10	10	3,7, and 14d	RCT	Pain, swelling, soft tissue healing
Al-Hamed/2017 [16]	Egypt	13 males and 34 females	25.24 ± 7.04Y	25	25	2,3,4,5,6, and 7d	RCT	Pain, alveolar osteitis, soft tissue healing
Gülşen/2017 [21]	Turkey	21 males and 9 females	20.03Y	30	30	1,2,3, and 7d	RCT, split-mouth	Pain

*Y* years, *d* days, *RCT* randomized controlled trial, *NA* Not available

**Table 2:** Summary of findings table

Impact of PRF on mandibular third molar surgery recovery						
Patient or population: patients with mandibular third molar surgery recovery Settings: outpatient Intervention: PRF Comparison: Non-PRF						
Outcomes	Illustrative comparative risks* (95% CI)		Relative effect (95% CI)	No of Participants (studies)	Quality of the evidence (GRADE)	Comments
	Assumed risk	Corresponding risk				
	Non-PRF	PRF				
Pain Visual analog scale Follow-up: 1-7 days	The mean pain in the control groups was 7.52	The mean pain in the intervention groups was 0.53 standard deviations lower (1.02 to 0.05 lower)		322 (6 studies)	⊕⊕⊝⊝ low^1,2^	
Swelling A flexible ruler Follow-up: 1-7 days	The mean swelling in the control groups was 20.79	The mean swelling in the intervention groups was 0.55 standard deviations lower (1.08 to 0.01 lower)		212 (4 studies)	⊕⊕⊕⊝ moderate^3^	
Trismus Measuring the distance Follow-up: 1-7 days	The mean trismus in the control groups was 24.35	The mean trismus in the intervention groups was 0.09 standard deviations lower (0.68 lower to 0.5 higher)		131 (4 studies)	⊕⊝⊝⊝ very low^3,4,5^	
Alveolar osteitis Follow-up: 2-90 days	179 per 1000	63 per 1000 (29 to 134)	RR 0.35 (0.16 to 0.75)	246 (3 studies)	⊕⊕⊝⊝ low^1,5^	
Osteoblastic activity Follow-up: 28-90 days	The mean osteoblastic activity in the control groups was 4.29	The mean osteoblastic activity in the intervention groups was 0.05 higher (0.44 lower to 0.55 higher)		68 (2 studies)	⊕⊝⊝⊝ very low^1,2,5^	
Soft tissue healing Follow-up: 2-14 days		The mean soft tissue healing in the intervention groups was 1.03 higher (0.32 lower to 2.38 higher)		70 (2 studies)	⊕⊕⊝⊝ low^1,4^	

*The basis for the assumed risk (e.g. the median control group risk across studies) is provided in footnotes. The corresponding risk (and its 95% confidence interval) is based on the assumed risk in the comparison group and the relative effect of the intervention (and its 95% CI)

*CI* Confidence interval, *RR* Risk ratio;

GRADE Working Group grades of evidence

High quality: Further research is very unlikely to change our confidence in the estimate of effect

Moderate quality: Further research is likely to have an important impact on our confidence in the estimate of effect and may change the estimate

Low quality: Further research is very likely to have an important impact on our confidence in the estimate of effect and is likely to change the estimate

Very low quality: We are very uncertain about the estimate

^1^ Having non-blinded study

^2^ The significant heterogeneity

^3^ No allocation concealment

^4^ Risk of bias

^5^ Pooled results included no effects

### Quantitative synthesis

**Fig. 3:**
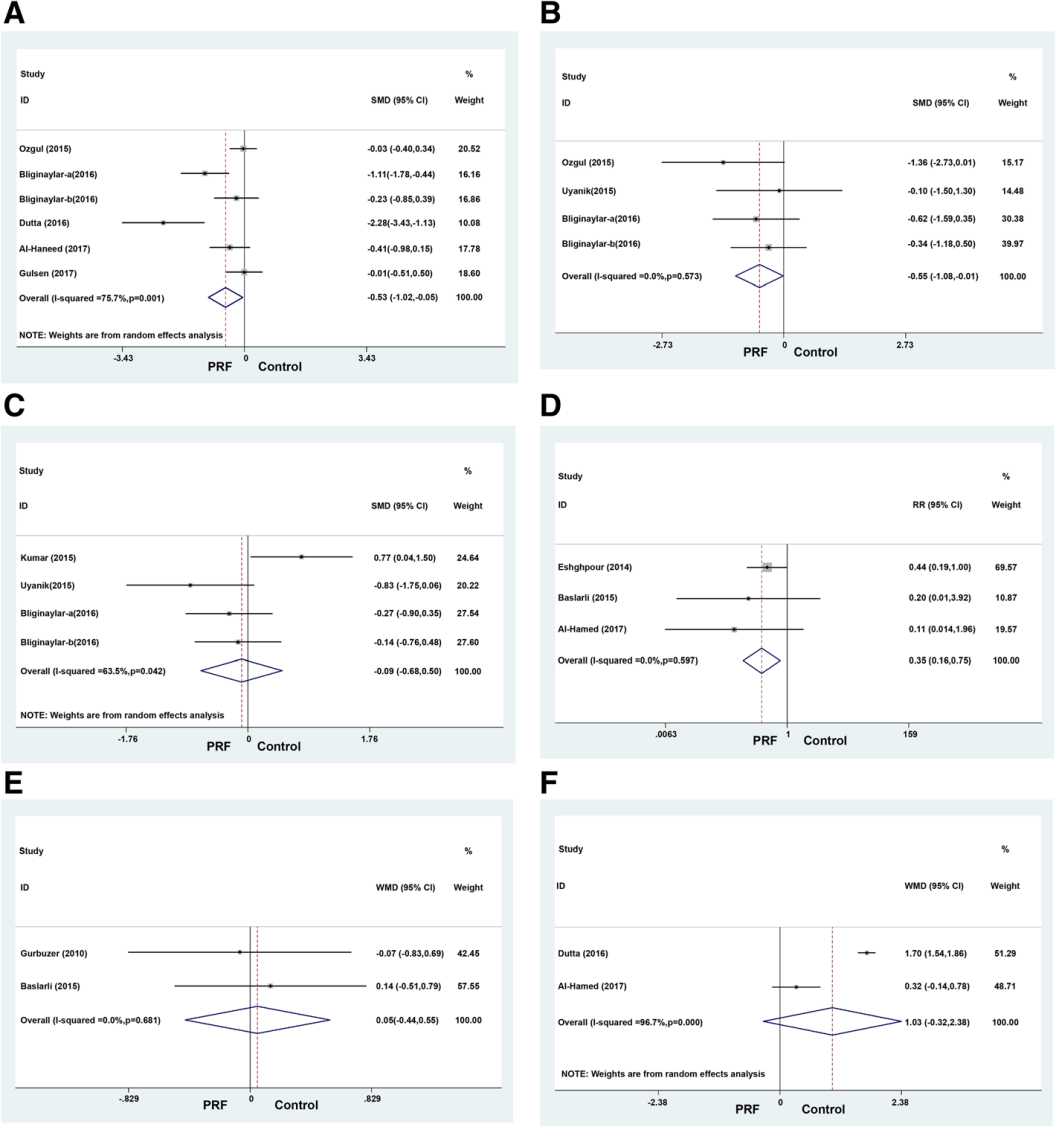
Forest plots showing the effect of PRF vs. control after mandibular third molar surgery. **a** Pain; **b** Swelling; **c** Trismus; **d** Alveolar osteitis; **e** Osteoblastic activity; **f** Soft tissue healing

*Postoperative pain*: The 6 studies [19–21, 32–34] that provided the outcomes regarding the postoperative pain in patients, who received PRF and control treatments, were included in the meta-analysis. A significant difference was observed in the postoperative pain on the third day (SMD = − 0.53, 95% CI: − 1.02 to − 0.05, *P*_heterogeneity_ = 0.001, *I*^*2*^ = 75.7%) between the two groups (Fig. 3a); however, no significant difference was noted on the first day (SMD = − 0.38, 95% CI: − 1.01–0.24, *P*_heterogeneity_ = 0.001, *I*^*2*^ = 82.1%) and seventh day (SMD = − 1.05, 95% CI: − 2.14–0.03, *P*_heterogeneity_ < 0.001, *I*^*2*^ = 90.3%). To explore the possible sources of heterogeneity, we conducted subgroup analyses according to measuring method. The results are summarized in Table 3. Furthermore, the between-study heterogeneity within subgroups remained substantial in most analyses.

**Table 3:** Subgroup analysis of the meta-analysis

Outcomes	Subgroup	Number of trials	Effect (95% CI)	Estimate for overall effect	Heterogeneity
Pain	Total(1 day)	4	−0.38(−1.01,0.24)	*P* = 0.231	*I*^2^ = 82.1%, *P* = 0.001
	VAS(1 day)	3	−0.59 (−1.45, 0.27)	*P* = 0.181	*I*^2^ = 86.1%, *P* = 0.001
	VAS and VRS(1 day)	1	0.16 (− 0.35, 0.66)	*P* = 0.545	
	Total(3 day)	6	−0.53(−1.02,0.05)	*P* = 0.032	*I*^2^ = 75.7%, *P* = 0.001
	VAS(3 day)	5	−0.67 (−1.26, − 0.08)	*P* = 0.026	*I*^2^ = 78.6%, *P* = 0.001
	VAS and VRS (3 day)	1	−0.01 (− 0.51, 0.50)	*P* = 0.975	
	Total(7 day)	4	−1.05 (−2.14, 0.03)	*P* = 0.057	*I*^2^ = 90.3%, *P* < 0.001
	VAS(7 day)	3	− 1.62 (−3.63, 0.39)	*P* = 0.113	*I*^2^ = 93.2%, *P* < 0.001
	VAS and VRS (7 day)	1	0.07 (− 0.44, 0.58)	*P* = 0.786	
Trismus	Total	4	−0.19 (− 0.88, 0.50)	*P* = 0.596	*I*^2^ = 73.1%, *P* = 0.011
	Ustun method	3	−0.46 (− 0.99, 0.07)	*P* = 0.088	*I*^2^ = 39.7%, *P* = 0.190
	Other	1	0.77 (0.04, 1.50)	*P* = 0.039	

*VAS* visual analogue scale, *VRS* verbal scale

### Postoperative swelling

The 4 studies [19, 32–34] that provided outcomes regarding the postoperative swelling in patients, who received PRF and control treatments, were included in the meta-analysis. A significant difference was observed in the postoperative swelling on the first day (WMD = − 0.55, 95% CI: − 1.08 to − 0.01, *P*_heterogeneity_ = 0.573, *I*^*2*^ = 0) between the two groups (Fig. 3b); however, no significant difference was observed on the third day (WMD = − 1.00, 95% CI: − 2.17–0.17, *P*_heterogeneity_ < 0.001, *I*^*2*^ = 94.8%) and seventh day (WMD = − 0.61, 95% CI: − 1.32–0.10, *P*_heterogeneity_ = 0.046, *I*^*2*^ = 74.9%).

### Trismus

This outcome was reported in 3 trials [31, 33, 34] that compared PRF to the control treatments. Any significant difference was not observed in the trismus on the first day (SMD = − 0.19, 95% CI: − 0.88–0.50, *P*_heterogeneity_ = 0.011, *I*^*2*^ = 73.1%) (Fig. 3c), third day (SMD = − 0.25, 95% CI: − 0.64–0.15, *P*_heterogeneity_ = 0.491, *I*^*2*^ = 0), and seventh day (SMD = − 0.25, 95% CI: − 0.64–0.15, *P*_heterogeneity_ = 0.764, *I*^*2*^ = 0) between the two groups. To explore the possible sources of heterogeneity, we conducted subgroup analyses according to measuring method. The results are summarized in Table 3. Furthermore, the between-study heterogeneity within subgroups was significantly reduced.

### Alveolar osteitis

The outcome was reported in 3 trials [20, 29, 30], and a fixed effects model did not reveal any significant heterogeneity between the studies. However, a significant difference was observed in the incidence of alveolar osteitis (RR = 0.35, 95% CI: 0.16–0.75, *P*_heterogeneity_ = 0.597, *I*^*2*^ = 0%) between the two groups (Fig. 3d).

### Osteoblastic activity

This outcome was reported in 2 trials [28, 30] that compared PRF to the control treatments. No significant heterogeneity was found between the studies as assessed by the fixed effects model. Also, no significant difference was observed in the osteoblastic activity (WMD = 0.05, 95% CI: − 0.44–0.55, *P*_heterogeneity_ = 0.681, *I*^*2*^ = 0%) between the two groups (Fig. 3e).

### Soft tissue healing

This outcome was reported in 2 trials [19, 20]. A significant heterogeneity occurred between the two studies as evaluated by the random effects model. However, no significant difference was observed in the soft tissue healing (WMD = 1.03, 95% CI: − 0.32–2.38, *P*_heterogeneity_ < 0.001, *I*^*2*^ = 96.7%) between the two groups (Fig. 3f).

### Sensitivity analysis

**Fig. 4:**
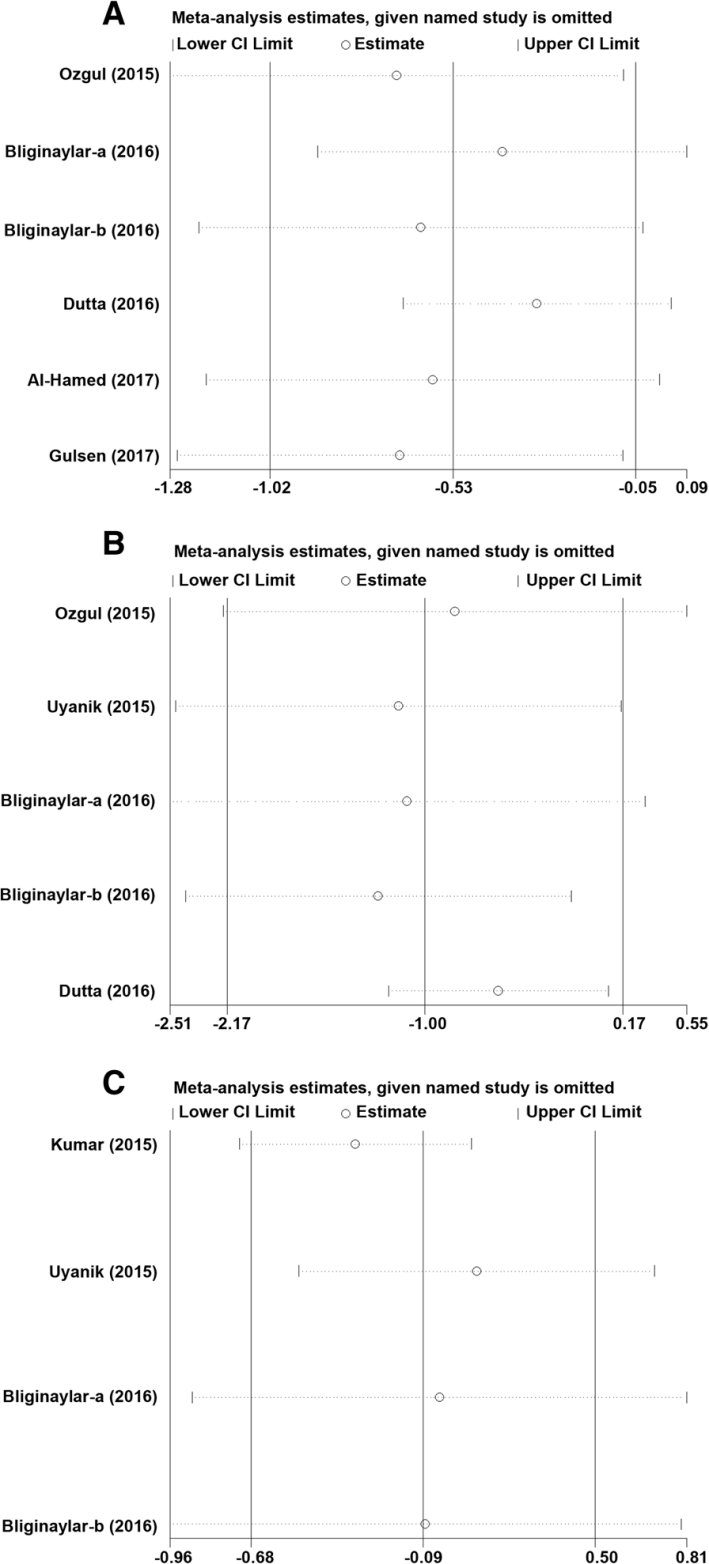
Sensitivity analysis of the effect of PRF vs. control after mandibular third molar surgery. **a** Pain; **b** Swelling; **c** Trismus

Sensitivity analyses were performed to assess the influence of individual dataset on the pooled estimate by sequential removal of each eligible study. However, the overall statistical significance did not change, indicating the robustness of the current results (Fig. 4).

### Publication bias

**Fig. 5:**
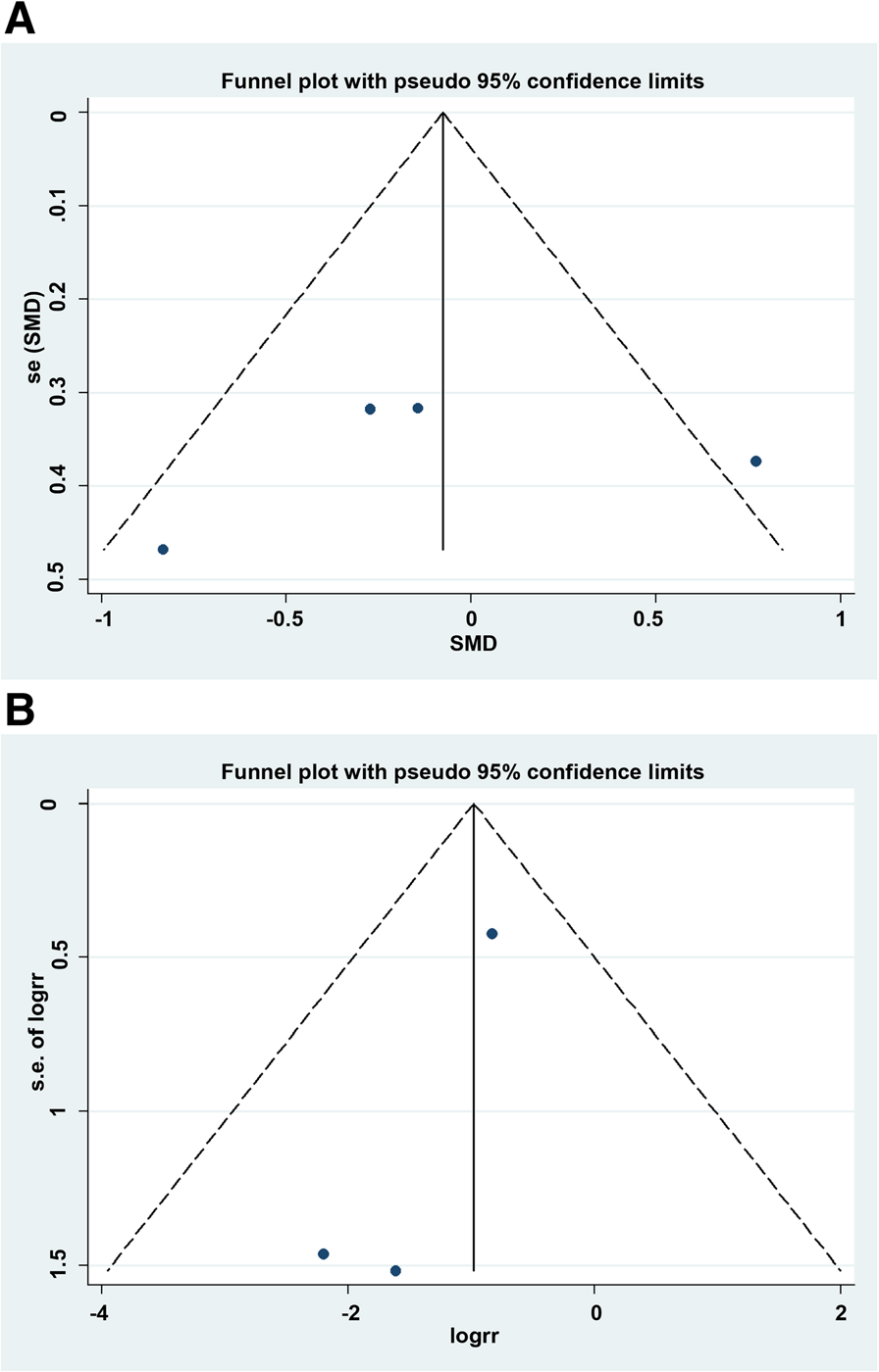
Funnel plot for publication bias test. Each point represented an independent study for the indicated association. **a** Trismus; **b** Alveolar osteitis

Finally, the Egger’s regression test did not show any significant evidence of asymmetrical distribution in the funnel plot in trismus (Begg’s test *P* = 0.734; Egger’s test *P* = 0.677) and alveolar osteitis (Begg’s test *P* = 1.000; Egger’s test *P* = 0.198) (Fig. 5).

## Discussion

The physiological additives modulate the inflammation and increase the therapeutic effect postoperatively; the use of fibrin adhesives has been documented in the past three decades [12–14]. However, due to the risk of cross-infection and cumbersome protocols for preparation, the use of these additives has been controversial. The present systematic review and meta-analysis was conducted to assess the effect of PRF on the healing process of the alveolar socket after surgical extraction of the mandibular third molars. The current results showed a beneficial effect of PRF in relieving pain and swelling and reducing the incidence of alveolar osteitis after the extraction of an impacted lower third molar. However, no statistically significant difference was observed between the two groups with respect to trismus, osteoblastic activity, and soft tissue healing. PRF is the second generation of platelet concentrates (PRP is the first generation). It is characterized by slow polymerization during preparation, which produces a fibrous protein network similar to the natural cells in order to enhance cell migration and proliferation. As a reservoir of platelets, cytokines, leukocytes, and immune cells, PRF allows a sustained release of cytokines such as VEGF, PDGF, TGF, and epidermal growth factor (EGF) that play a key role in vascular and tissue healing and scarring [11, 13, 14]. Reportedly, PRF also enhances angiogenesis, supports immunity, and increases the coverage of the injured tissue by enhancing the positive effects on epithelial cells and fibroblasts [11]. In oral and maxillofacial regions, PRF is widely used in simple graft or combination with allograft or xenograft [35]. In addition, the PRF clots are used for the flapless treatment of acute sinus perforations [36]. The extraction for socket preservation, intrabony defects, and periodontal problems are the other indications of PRF usage [11].

To the best of our knowledge, the current meta-analysis is the largest study investigating the impact of PRF on a mandibular third molar in 314 patients from 10 studies. Compared to the studies by Al-Hamed et al. [16] and Canellas et al. [17], we found that the local application of PRF, during the extraction of the lower third molar, significantly relieved pain on the postoperative third day and swelling on the postoperative first day by meta-analysis, while the previous studies did not perform a quantitative data synthesis because of the limited available data. Compared to the study by He et al. [18], we found that the local application of PRF, during the extraction of the lower third molar, significantly relieved the swelling on the postoperative first day, while the previous study indicated that PRF significantly relieved the postoperative swelling on the third day. This inconsistency in the result might be attributed to the newly identified eligible study. Heterogeneity is a potential issue when interpreting the results of meta-analyses, in which, heterogeneity was detected while analyzing the pain and soft tissue healing; thus, the random-effects model was used. Different study types, scales of measurement, time intervals, and surgical protocols are possible explanations for the heterogeneity. Furthermore, sensitivity analyses were also conducted by sequential exclusion of each eligible study. However, the pooled estimate did not alter significantly, thereby strengthening the conclusions.

Furthermore, the current meta-analysis also presented some limitations: First, the number of studies for some parameter analysis was small, which might lessen the statistical power. Second, the studies exhibited significant heterogeneity. Different study types, scales of measurement, time intervals, and surgical protocols are possible explanations for the heterogeneity. Third, bias could be introduced if studies published in a language other than English were excluded. Finally, the follow-up time varied considerably among the 10 studies, which ranged from 1 to 90 days and limited the assessment of long-term clinical effects of PRF on the mandibular third molar.

## Conclusions

In conclusion, despite the limitations of the meta-analysis, our study confirmed that PRF only reduces some of the postoperative complications but does not prevent them. PRF administered after third molar extraction significantly relieved pain, swelling, and reduced the incidence of alveolar osteitis. Therefore, further studies with a larger dataset and well-designed models are essential to validate the current findings.

## Additional file

Additional file 1: **Table S1.** Search strategies (DOC 29 kb)

## Data Availability

The datasets used and/or analyzed during the current study are available from the corresponding author on reasonable request.

## Abbreviations

CI: Confidence interval
EGF: Epidermal growth factor
PRF: Platelet-rich fibrin
RCTs: Randomized controlled trials
RR: Risk ratio
SMD: Standard mean difference
TGF: Transforming growth factor
VEGF: Vascular endothelial growth factor
WMD: Weighted mean difference
