# Supplementary material for: Impact of platelet-rich fibrin on mandibular third molar surgery recovery: a systematic review and meta-analysis
Source: BMC Oral Health. 2019 Jul 25;19:163. doi: 10.1186/s12903-019-0824-3 (PMC6659259; doi:10.1186/s12903-019-0824-3)
Supplement: Supplementary file 1 — Table S1. Search strategies (DOC 29 kb) [file 12903_2019_824_MOESM1_ESM.doc]

**Table S1 Search strategies.**

| **Database** | **Period of Search** | **Search strategies** |
| --- | --- | --- |
| PubMed | September 2017 | ("molar, third"[MeSH Terms] OR ("molar"[All Fields] AND "third"[All Fields]) OR "third molar"[All Fields] OR ("third"[All Fields] AND "molar"[All Fields])) AND ("platelet-rich fibrin"[MeSH Terms] OR ("platelet-rich"[All Fields] AND "fibrin"[All Fields]) OR "platelet-rich fibrin"[All Fields] OR ("platelet"[All Fields] AND "rich"[All Fields] AND "fibrin"[All Fields]) OR "platelet rich fibrin"[All Fields]) |
| Embase | September 2017 | (' third molar '/exp OR ' third molar ') AND (' platelet-rich fibrin 'OR 'PRF') AND ' oral surgery ' |
| Cochrane Library | September 2017 | ((third molar) OR MeSH descriptor third molar explode all trees) AND (platelet-rich fibrin OR PRF) AND oral surgery |
